# Heredity, Eugenics and Race Conservation

**Published:** 1912-05

**Authors:** R. H. L. Bibb


### EDITORIAL DEPARTMENT

DR. F. E. DANIEL, Editor DR. R. H. L. BIBB, Associate Editor

### HEREDITY, EUGENICS AND RACE CONSERVATION.

Ever since the Almighty declared Himself to be a jealous God, "visiting the iniquities of the fathers upon the children unto the third and fourth *generations*," man has been constantly warned that his children and his children's children would be punished for his individual iniquities, and yet it would seem that because of "the hardness of his heart and the reprobacy of his mind," his iniquitous ways have grown larger and larger with time, in spite of the multiplied proofs that this warning was neither an idle boast nor a "scare-crow" to frighten the wayward into submission and righteous living.

In like manner, from time immemorial—so remote that "the mind of man runneth not to the contrary"—the medical profession has warned mankind that certain diseases, predispositions, infirmities and characteristics—mental, moral and physical—were transmitted from parent to offspring, citing the repleted criminal and divorce-courts, penitentiaries, almshouses, jails, hospitals, insane asylums, bawdy houses, abortions and infant mortality, grog shops—the cesspools of human putresence—and the many wrecks in private households as confirmatory of its contentions; and yet, withal, sinful man has continued to "beget sons and daughters," and to give them in marriage with very much less concern than a careful breeder exercises in selecting a bull to impregnate a favorite heifer in his cowpens, with the result that the integrity of the human race is at a lower ebb and in greater danger today than ever before in the history of the world. So unmindful, indeed, have the heads of many families become that they actually create, accept and court conditions for their sons and daughters which they would not, for a moment, tolerate in their breeding pens, with the result that many of these matrimonies are childless, others a succession of abortions, others bear children too feeble to live, only to see them die in infancy, whilst yet others, after many trials and tribulations, succeed in raising their offspring—they do not develop—to adult age, miserable examples of neurasthenic runts, incapable of performing the duties pertaining to men and women, who live their short careers and die as they had lived—unsightly stigmata on the human family.

As illustrative of the transmission of the iniquities of fathers to their children, the experience of Dr. Martin W. Barr, Chief Physician of the Pennsylvania Training School for Feeble-minded Children, based on an experience of more than 4000 cases, as related in the February, 1912, issue of the *Alienist and Neurologist*, is reproduced here. The doctor writes of a father aged 38, with 19 defective children, he and his wife both under the normal mentally; another couple with 9 imbecile children; an idiot, mother of 7 idiot children, of a father with 2 daughters and an illegitimate grandchild, all feeble-minded; a family of 7 generations of 138 individuals, with 10 premature births, 16 insane, 7 imbeciles, 3 epileptics, 32 feeble-minded, and 80 apparently normal but helplessly slaves to a neurotic heredity; of 15 imbecile girls, 3 of whom were prostitutes, 9 had illegitimate children—1 had 2, 2 children being the result of incestous intercourse with brothers, 2 were epileptics, 7 had idiotic children; of 4 feeble-minded women who had 40 feeble-minded children; of a feeble-minded woman who had lived in an almshouse from early life who had 6 illegitimate children who inherited the maternal defect; of an imbecile drunkard, father of 3 feeble-minded children, 1 of whose daughters bore an illegitimate, feeble-minded child before she was 16 years old, one son was a harmless imbecile, the other a thief and a sexual pervert; of another family of three generations below the normal, father, mother, mother's sister and father's uncle imbeciles and 5 children feeble-minded; of an insane woman, whose brother and sister suicided, who had 5 sons, 1 feeble-minded, and a drunkard, 1 insane, who had 3 imbecile children, 1 epileptic, with 3 epileptic sons; 1 insane without offspring, and 1 apparently normal who had an imbecile son and an epileptic daughter.

This, it will be noted, is the experience of *one* physician in the State of Pennsylvania, where it is claimed there are 10,000 cases of "avowed imbecility," about one-third of whom are sequestrated. It is highly interesting to speculate as to what a combined report of the 3000 physicians of that State would show concerning the remainder of that imbecile population—the 6666, not sequestrated.

The history of Max Jukes and his family has become notorious, and is, in itself, sufficient to show it to be the *imperative duty* of man to take measures, by *vasectomy* for the male, and *tubesectomy* for the female, as being equally efficient and less dangerous than *castration,* besides being free of constitutional objections, to prevent a repetition of that unsightly blotch. Jukes, it appears, was born in 1720. He was a worthless, drunken vagabond; 300 of his descendants died in hospitals; 300 died in infancy; 440 suffered venereal diseases; 400 suffered, in various ways, from iniquitous living; 50 were notoriously immoral; 60 spent an average of twelve years in prison; 7 were murderers and 130 were punished, more or less frequently, for lesser crimes and misdemeanors. So far as is known, none of Jukes' descendants contributed anything, in any way, to the welfare of either the State or society, and it is estimated that the family cost the State (New York) over *a million and a half of dollars.*

Fortunately, however, good, as well as bad, qualities—and this wise provision of nature has done more to protect racial integrity than have the combined efforts of man—are also handed down "unto children of the third and fourth *generations,*" as is most beautifully illustrated in the family of Jonathan Edwards. This noted expounder of the physical-hell theory of theology—"a bottomless pit filled with fire and brimstone"—whose teachings weighed like a mighty incubus over the heads of old and young for several generations, was born in Connecticut in 1703. In 1900 it is reported that researches brought to light the histories of 1394 of his descendants. Thirteen were university presidents and 64 were university professors; 100 were clergymen; 75 officers in the army; 60 were physicians; 180 were judges, or lawyers; 80 were in the civil service; 3 were United States Senators; 1 was Vice-President of the United States; 1 was president of a large navigation company, whilst many were Governors of States, members of Congress, mayors of cities and plenipotentiaries. Thirty-three States, ninety-two cities and several foreign countries profited by the beneficent activity of the Edwards family; and it is not recorded that a single member of this family was ever under the ban of the law for even the most trivial offense.

In every community there are many degenerates, semi-insane, imbeciles, consumptives, drunkards, syphilitics and sexual perverts enjoying the fullest liberty of action, who are daily polluting the race with their degenerate offspring. It is against a continuation of this heinous crime upon race integrity that the profession voices its solemn protest and calls upon every lover of mankind to unite with it in averting the terrible doom that hangs over the race. For years this protest has fallen, like a wail in the wilderness, upon hears that heard not, or, hearing, heeded not, until within the recent past, when the newspapers and clergy began to take heed, and, as a result of the combined effort of this triune, the medical profession, the newspapers and the pulpit, seven States have passed laws for the purpose of cutting off the production of the insane and criminals; and some of the leading churches in

Chicago have given notice that after April 1st their clergy will marry *only those who present a certificate from a reputable physician that the candidates for matrimony are free from defects, taints and diseases that may be transmitted from parents to their children.*

In addition, the leading newspapers throughout the world are beginning to use their editorial columns in the interest of eugenics and racial conservation. The subjects—"Health and Marriage," "National Mental Hygiene" and "New Science Eugenics"—are treated of in highly creditabe editorials in recent issues of the *Austin Statesman*, in one of which it is stated that "this new work—eugenics—is being taken up here in Texas, and will be pushed to success." In this connection, why may not "this new science" find a place in the University curriculum? Or, failing in that: why not invite noted expounders of eugenics to give a series of lectures, from time to time, in the lecture halls of that great institution? Surely the subject is of sufficient importance, present and future; of equal importance, at least, with the life, writings and teachings of the Russian count so recently recounted to its professors and alumni. Eugenics should be taught in all the schools, colleges and universities in the State as a *prescribed study*. Incidentally, it is well taught by every professor in the Medical Department at Galveston, and were it only taught one-half as well in the other schools of the State, in a few years it would create such an interest in its favor that the Legislature would hasten to enact laws that would not only restrict, to a minimum, the present wholesale pollution of its population, but such as would, also, be a faithful reflex of the culture and forethought of the people of the great State of Texas.

In this connection, it may not be out of place to note the fact that the Federal Congress, *after five years of travail,* has very recently passed a bill, which the President *will probably sign,* creating a "Children's Bureau"—which, sad to write, in the absence of the department provided for in the Owen bill—"in the Department of Commerce and Labor." This Bureau will be charged with "the collection and dissemination of data relating to all phases and conditions of child-life, infant mortality, birth rate, orphanage, etc. This is all very good, as far as it goes, even if it is done along with the same investigations into conditions governing the welfare, or otherwise, of jack rabbits, bantam chickens and bull frogs.

If Lord Beaconfield's dictum, "The care of the public health is the first duty of a statesman," is true, then it would be proper for our Senators, Congressmen and legislators to take notice. Selah!

R. H. L. BIBB.

